# Impact of Ambient Humidity on Child Health: A Systematic Review

**DOI:** 10.1371/journal.pone.0112508

**Published:** 2014-12-12

**Authors:** Jinghong Gao, Yunzong Sun, Yaogui Lu, Liping Li

**Affiliations:** 1 Injury Prevention Research Center, Shantou University Medical College, No. 22 Xinling Road, Shantou, Guangdong, 515041, China; 2 Department of Public Health, Shantou University Medical College, No. 22 Xinling Road, Shantou, Guangdong, 515041, China; Alberta Provincial Laboratory for Public Health/University of Alberta, Canada

## Abstract

**Background and Objectives:**

Changes in relative humidity, along with other meteorological factors, accompany ongoing climate change and play a significant role in weather-related health outcomes, particularly among children. The purpose of this review is to improve our understanding of the relationship between ambient humidity and child health, and to propose directions for future research.

**Methods:**

A comprehensive search of electronic databases (PubMed, Medline, Web of Science, ScienceDirect, OvidSP and EBSCO host) and review of reference lists, to supplement relevant studies, were conducted in March 2013. All identified records were selected based on explicit inclusion criteria. We extracted data from the included studies using a pre-designed data extraction form, and then performed a quality assessment. Various heterogeneities precluded a formal quantitative meta-analysis, therefore, evidence was compiled using descriptive summaries.

**Results:**

Out of a total of 3797 identified records, 37 papers were selected for inclusion in this review. Among the 37 studies, 35% were focused on allergic diseases and 32% on respiratory system diseases. Quality assessment revealed 78% of the studies had reporting quality scores above 70%, and all findings demonstrated that ambient humidity generally plays an important role in the incidence and prevalence of climate-sensitive diseases among children.

**Conclusions:**

With climate change, there is a significant impact of ambient humidity on child health, especially for climate-sensitive infectious diseases, diarrhoeal diseases, respiratory system diseases, and pediatric allergic diseases. However, some inconsistencies in the direction and magnitude of the effects are observed.

## Introduction

“Warming of the climate system is unequivocal”, according to the United Nations Intergovernmental Panel on Climate Change, and is expected to continue for the next several decades [1]. Even if the concentrations of all greenhouse gases and aerosols remain constant at year 2000 levels, a further warming of about 0.1°C per decade would be expected [1], [2].

Climate warming will change rainfall patterns, and increase humidity and risk of floods, although a geographical and temporal heterogeneity in the occurrence of these changes is expected [3], [4]. There is strong consensus among expert scientists that with climate change, meteorological factors (e.g. temperature, humidity) are affecting and will increasingly influence human health [5]–[8]. Humidity along with other weather factors can affect the incidence and distribution of infectious diseases by influencing the reproduction, development and population dynamics of vectors [9], [10]. Increasingly warm and humid climates have been observed to be associated with increasing atmospheric levels and density of aeroallergens, indicating that ambient humidity may indirectly affect the incidence and prevalence of allergic diseases [11], [12]. In addition, humidity also plays a great role in the transmission of viral diseases, such as influenza [7], [13].

Compared with adults, children are inherently sensitive to climate change because they are physiologically and metabolically less effective at adapting to ambient humidity and other weather-related exposures. Their relatively immature immune systems increase the risk of serious consequences from a variety of infectious diseases [14]. In 2000, the World Health Organization estimated that climate change contributed to more than 150,000 deaths and 5.5 million lost disability-adjusted life years worldwide and more than 88% of this burden occurs in children under the age of 5 years [15]. According to a 2012 report of the World Health Organization, pneumonia, diarrhoeal disease and malaria, all of which are climate-sensitive diseases, are three of the leading causes of death among children under age five. For example, malaria is a climate-sensitive illness to which children are particularly vulnerable, currently causing 350–500 million illnesses annually, and more than 1 million deaths, with children experiencing disproportionately high levels of both morbidity and mortality (75% of malaria deaths occur in children younger than 5 years of age) [16].

However, although pediatric health care professionals have written about the effects of climate change on child health, relatively limited research has been conducted specifically on the impact of ambient humidity on child health. To our knowledge, almost all of the studies investigating the association between climate change and child health mainly focus on temperature or rainfall, while ambient humidity is usually considered as a dispensable and alternative variable.

Additionally, to date, no systematic review has specifically focused on the relationship between ambient humidity and child health. In order to fill this knowledge gap, the primary objective of this review is to improve our understanding of how humidity, both directly and indirectly, can influence child health and to promote additional research in this field by presenting and summarizing the findings of all relevant studies related to the impact of ambient humidity on child health.

## Methods

### Data sources and search strategy

Relevant peer-reviewed journal databases (PubMed, Medline, Web of Science, ScienceDirect (Elsevier), OvidSP and EBSCO host) were searched using the following MeSH (Medical Subject Headings) terms and keywords: “climate change”, “humidity”, “wet”, “dew point”, “child”, “infant”, “adolescent” and “pediatric”. The search terms and keywords were adapted to suit the requirements of each database to facilitate a comprehensive search. After selection of the full text, the references of the identified articles were reviewed and the necessary hand search was performed to recover potentially relevant studies not included in the major databases.

The search sought to identify journal articles published in English up to March 2013. In accordance with *the Convention on the Rights of the Child* proclaimed by the United Nations, we defined children as the group of people 0–18 years of age [17].

### Inclusion criteria

The following eligibility criteria were used in this study:

- Types of studies: the present review only included peer-reviewed original articles. Thus, reviews, books, meta-analyses and conference abstracts were excluded.
- Target population: only the studies involving children between 0–18 years of age were considered.
- Research factors: only studies in which humidity was one of the exposure indicators of interest were considered.
- Outcome measures: the reported results of the included studies should have included at least one quantitative effect estimate associated with humidity. An outcome measure was reported as either: percent change, regression coefficient, correlation coefficient, relative risk or odds ratio. We also required *P*-values or 95% confidence intervals (CIs) to have been reported.

### Study selection and data extraction

Three of the authors (Gao, Sun and Lu) independently assessed the potential relevance of all publications, identified during the database search, based on the information provided in the titles and abstracts. When reviewers disagreed, a discussion was held to obtain consensus. After screening by titles and abstracts, the full texts of the remaining articles were then obtained and critically reviewed by the first author.

We extracted data, from the final selected references, using a pre-designed data extraction form. The quantitative estimates of each study that met the eligibility criteria were carefully retrieved and the following key characteristics were captured: study region, target population, study design, humidity exposure variable, health outcomes and adjustment of confounders. All data extraction was performed by the first author (Gao) and cross-checked by another researcher (Sun). Any errors identified were corrected through discussion among all authors.

### Quality assessment

To date, the use of quality assessment tools to appraise observational studies included in systematic reviews is less well established than in reviews of randomized controlled trials, and there is no existing generally accepted quality assessment tool for observational and cross-sectional studies [18], [19]. Therefore, the STROBE method (Strengthening the Reporting of Observational Studies in Epidemiology), a guideline for reporting observational studies, was selected and adapted for our purpose to describe the reporting quality of the final identified articles [20], [21].

As in a previous systematic review, an adapted version of the STROBE checklist (**Table 1**) was used to create a binomial scoring system (e.g. criterion met  = 1, criterion not met  = 0). Final score percentages were based on the number of reporting quality criteria met divided by the number of criteria that were relevant during the quality assessment [22]. During this process, studies that controlled for potential confounding factors and included an outcome measure, such as relative risk, odds ratio or regression coefficient, were given higher quality ratings. Meanwhile, studies were given lower quality ratings if they did not include adjustment of confounders or only included outcome measures such as an estimated slope coefficient from simple linear regression or a correlation coefficient for which only the *P*-value was given. As no accepted gold standard exists for judging the quality of observational studies, in the present review we did not exclude any identified studies based on the quality assessment, which used an adapted version of the STROBE checklist.

**Table 1:** Adapted STROBE Statement—checklist of items that should be included in reports of observational studies (including additions/adaptations for accommodating meteorological data).

	Item No	Recommendation
**Title and abstract**	1	(*a*) Indicate the study's design with a commonly used term in the title or the abstract
		(*b*) Provide in the abstract an informative and balanced summary of what was done
		and what was found
**Introduction**		
Background	2	Explain the scientific background and rationale for the investigation being reported
Objectives	3	State specific objectives, including any pre-specified hypotheses
**Methods**		
Study design	4	Present key elements of study design early in the paper
Setting	5	Describe the setting, locations, and relevant dates, including periods of recruitment,
		exposure, follow-up, and data collection
Participants	6	(*a*) *Cohort study*—Give the eligibility criteria, and the sources and methods of
		selection of participants. Describe methods of follow-up
		*Case-control study*—Give the eligibility criteria, and the sources and methods of case
		ascertainment and control selection. Give the rationale for the choice of cases and
		controls
		*Cross-sectional study*—Give the eligibility criteria, and the sources and methods of
		selection of participants
		(b) *Cohort study*—For matched studies, give matching criteria and number of
		exposed and unexposed
		*Case-control study*—For matched studies, give matching criteria and the number of
		controls per case
Variables	7	Clearly define all meteorological variables, outcomes, exposures, predictors, potential
		confounders, and effect modifiers. Give diagnostic criteria, if applicable
Data sources	8*	For each variable of interest, especially meteorological and outcome variables, give
		sources of data and details of methods of assessment (measurement). Describe
		comparability of assessment methods if there is more than one group
Bias	9	Describe any efforts to address potential sources of bias
Study size	10	Explain how the study size was arrived at
Quantitative variables	11	Explain how quantitative meteorological and outcome variables were handled in
		the analyses, including how meteorological variables were handled before using for
		analysis, and what kind of statistical software(s), as well as its/their version number
		were used to complete related statistical analysis. If applicable, describe which
		groupings were chosen and why
Statistical methods	12	(*a*) Describe all statistical methods, including those used to control for confounders
		(*b*) Describe any methods used to examine meteorological and outcome subgroups
		and interactions
		(*c*) Explain how missing data of meteorological variable and outcome were addressed
		(d) *Cohort study*—If applicable, explain how loss to follow-up was addressed
		*Case-control study*—If applicable, explain how matching of cases and controls was addressed
		
		*Cross-sectional study*—If applicable, describe analytical methods taking account
		the sampling strategy
		(e) Describe any sensitivity analyses
**Results**		
Participants	13*	(a) Report numbers of individuals at each stage of the study—e.g. numbers potentially
		eligible, examined for eligibility, confirmed eligible, included in the study, completing
		follow-up, and analysed
		(b) Give reasons for non-participation at each stage
		(c) Consider use of a flow diagram
Descriptive data	14*	(a) Give characteristics of study participants (e.g. demographic, clinical, social) and
		information on exposures and potential confounders. Summarize meteorological
		characteristics of the study area (if applicable)
		(b) Indicate the number of participants with missing data for each variable of interest,
		especially meteorological and outcome variables
		(c) *Cohort study*—Summarize follow-up time (e.g. average and total amount)
Outcome data	15*	*Cohort study*—Report numbers of outcome events or summary measures over time
		*Case-control study*—Report numbers in each exposure category, or summary
		measures of exposure
		*Cross-sectional study*—Report numbers of outcome events or summary measures
Main results	16	(*a*) Give unadjusted estimates and, if applicable, confounder-adjusted estimates and
		their precision (e.g. 95% confidence interval). Make clear which confounders were
		adjusted for and why they were included
		(b) Report category boundaries when meteorological or continuous variables were
		categorized
		(c) If relevant, consider translating estimates of relative risk into absolute risk for a
		meaningful time period
Other analyses	17	Report other analyses done—e.g. correlation analysis, analyses of subgroups and
		interactions, and sensitivity analyses
**Discussion**		
Key results	18	Summarise key results with reference to study objectives
Limitations	19	Discuss limitations of the study, taking into account sources of potential bias or
		imprecision. Discuss both direction and magnitude of any potential bias
Interpretation	20	Give a cautious overall interpretation of results considering objectives, limitations,
		multiplicity of analyses, results from similar studies, and other relevant evidence
Generalizability	21	Discuss the generalizability (external validity) of the study results
**Other information**		
Funding	22	Give the source of funding and the role of the funders for the present study and, if
		applicable, for the original study on which the present article is based

*Give information separately for cases and controls in case-control studies and, if applicable, for exposed and unexposed groups in cohort and cross-sectional studies.

### Data synthesis

Due to heterogeneity in the target populations, outcome measures and design of studies, we did not conduct an overall summary estimate (e.g. meta-analysis). Instead, we summarized the evidence in a descriptive synthesis.

## Results

**Figure 1:**
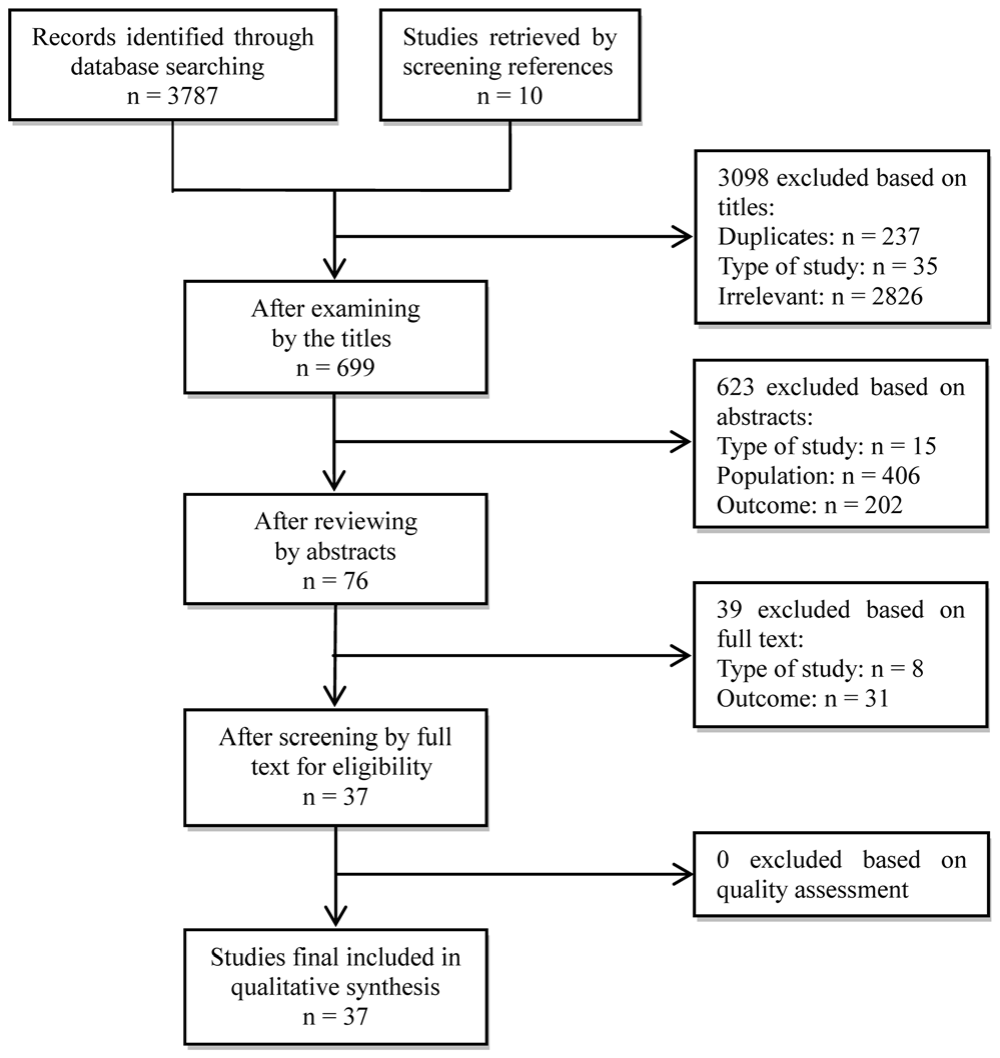
Flow chart of the screening and selection process of the study articles.

A total of 3787 records were identified from the electronic database searches, and 10 additional studies were retrieved by reviewing references. After screening by titles and abstracts, 76 articles were selected for examination of the full text. Finally, 37 papers remained for inclusion in this systematic review (**Fig. 1**). Then quality assessment and data synthesis were performed to obtain the key results presented and summarized in **Table 2**. Among the 37 studies, 24% (9/37) involved infectious diseases, 32% (12/37) were on respiratory system diseases, and 35% (13/37) were focused on asthma and other allergic diseases. Nearly 73% (27/37) of the studies were conducted in high-income countries or regions. According to the adapted STROBE checklist, the reported quality scores ranged from 53.33% to 88.89%, with the majority of records (29/37) having scores above 70%, indicating a relatively high reporting quality of the articles. Studies using time-series analysis, Poisson regression or negative binomial regression were usually given higher scores.

**Table 2:** Characteristics of included studies about ambient humidity and children's health (n = 37).

Author(year)^a^	Study region	Target population	Study design and	Humidity	Target outcome	Statistical indicator	Adjustment for	Reporting
	and period	(number, age)	analysis method	exposure		and effect estimate	confounding	quality(%
				variable			factors	score)
YÜKSEL et al.^b^ (1996) [47]	Izmir, Turkey	21 children	Descriptive study,	Daily	Total daily	r = 0.578	No adjustments	53.33
	1993–1994	aged 5–14	Correlation and	relative	complaints of the	*P*<0.01	were conducted	
		years	variance analysis	humidity	asthma patient			
Guo et al.^b^ (1999) [48]	Taiwan	331,686	Descriptive study,	Daily	Asthma	Boys: R^2^ = 0.57, β = 0.37	Adjusted for age,	64.29
	1995–1996	children aged	Logistic regression	winter	prevalence rate	(95% CI: 0.0078–0.73,	history of atopic	
		0–15 years	analysis	month		*P*<0.05)	eczema and parental	
				humidity			education	
Checkley et al.^b^ (2000) [31]	Lima, Peru,	57 331 children	Time-series study,	Daily mean	Daily hospital	RR = 0.97	No adjustments	71.43
	1993–1998	under 10 years	Poisson GAM	relative	admissions for	(95% CI: 0.97–0.98)	were conducted	
				humidity	diarrheal diseases			
					per 1% increase in			
					relative humidity			
					at 37 lag days			
Weiland et al.^b^ (2004) [53]	Western	Children aged	Multilevel linear	Monthly mean	Prevalence rates	1) 13–14 years old children :	Adjusted for gross	60.00
	Europe	6–7 and 13–14	regression	lowest relative	of symptoms of	β = 0.13 (95% CI:0.04–0.23)	national product per	
	1992–1996	years	analyses	humidity	asthma	2) 6–7 years old children :	capita (GNP)	
						β = 0.09 (95% CI:0.02–0.15)		
Hashimoto et al.^b^ (2004) [55]	Tokyo, Japan	5559 children	Multiple logistic	Daily mean	Number of emergent	β = 0.075,	Adjusting for	74.07
	1998–2002	aged 2–15 years	regression analyses	relative	visits for childhood	(95% CI: 0.06–009,	calendar month and	
				humidity	asthma	*P*<0.001)	day of the week	
Viegas et al.^b^ (2004) [33]	Buenos Aires,	18561 children	Spearman's rank	Monthly mean	Respiratory viruses	1) RSV: r = 0.6, *P*<0.0001;	No adjustments	62.96
	Argentina	aged under 5	correlation test	relative	frequencies among	2) IA: r = 0.47, *P* = 0.0068	were conducted	
	1998–2002	years		humidity	the patients			
Lapeña et al.^b^ (2005) [34]	Leon, Spain.	167 children	Case-control study,	Weekly	Admission with lower	1) r = −0.15, *P* = 0.045;	No adjustments	72.41
	1995–2000	aged under 2	Correlation analysis	relative	respiratory tract	2) OR = 0.96 CI: 0.92–0.99)	were conducted	
		years	Multivariate logistic	humidity	infection due to RSV	*P*<0.01(absolute humidity		
			regression model			was not included in the model)		
Priftis et al.^b^ (2006) [44]	Athens,	18950 children	Multiple regression	Monthly mean	Monthly hospital	β = 1.106, *P*<0.05,	No adjustments	75.86
	Greece	aged 0–4 years	analysis	relative	admission rates for	r^2^ = 0.592	were conducted	
	1978–2000			humidity	childhood asthma			
Al-Toum et al.^b^ (2006) [35]	Amman,	200 Children	Descriptive study,	Monthly mean	Prevalence of RSV	r = 0.66, *P*<0.05	No adjustments	62.96
	Jordan	aged under 2	Correlation analysis	relative			were conducted	
	2002–2004	years		humidity				
Xirasagar et al.^b^ (2006) [52]	Taiwan	27275 Children	Cross-sectional study,	Monthly mean	Monthly asthma	1) Children aged 2–5 years:	Seasonality trend	70.97
	1998–2001	aged 0–14 years	ARIMA models,	relative	admission rates	r = −0.290, *P*<0.01	was evaluated	
			Spearman rank	humidity		2) Children aged 6–14 years:		
			correlations analysis			r = −0.415, *P*<0.001		
Yé et al.^b^ (2007) [28]	Kossi,	676 children	Descriptive study,	Daily mean	Incidence of clinical	β = −23.2151	Site of the study,	75.86
	Burkina Faso	aged 6–59	Logistic regression	relative	malaria	(95% CI: −36.7151 to	sex; age; presence	
	2003–2004	months		humidity		−9.7152)	of animal were	
							considered	
Kurt et al.^b^ (2007) [45]	Turkey	25843 children	Cross-sectional	Yearly mean	Allergic diseases	1) Asthma: OR = 1.009	Adjusted for age,	78.57
	2004	aged 6–15 years	study, and	relative		(95% CI: 1.002–1.015)	rural/urban sex,	
			Multivariate logistic	humidity		2) Wheezing: OR = 1.01	residence and	
			regression analysis	>70%		(95% CI: 1.003–1.02)	sibling number	
						3) Allergic rhinitis: OR =		
						1.006 (95% CI: 1.001–1.01)		
D'Souza et al.^b^ (2008) [32]	3 Australian	Children aged	Time–series study	Weekly mean	Weekly admissions	1) Canberra: IRR = 0.98	Adjusted for trend,	76.00
	cities	0–5 years	and log-linear	relative	for rotavirus diarrheal	(95% CI: 0.97–0.99)	season and previous	
	1993–2003		regression model	humidity	per 1% increase in	2) Brisbane: IRR = 0.98	2 weeks' admission	
					relative humidity	(95% CI: 0.97–0.99)		
					of the previous week	3) Melbourne: IRR = 0.99		
						(95% CI: 0.99–1.00)		
Omer et al.^b^ (2008) [36]	Lombok	2878 children	Negative binomial	Daily mean	Daily number of	IRR = 1.06 , *P*<0.001	No adjustments	85.71
	island,	aged 0–2 years	regression and	relative	RSV cases 8 days	(95% CI: 1.03–1.10)	were conducted	
	Indonesia		multivariate	humidity	later			
	2000–2002		regression					
Noyola. D. E. and Mandeville, P. B^b^ (2008) [38]	San Luis	1393 children	Regression	Weekly mean	Weekly number	β = 0.035, *P* = 0.0027	Temperature, hours	61.29
	Potosí,	aged 0–18	analysis	relative	of RSV cases	(95% CI: 0.012–0.058)	light and precipitation	
	Mexico.	years		humidity at			were considered	
	2002–2006			08:00 hours			in the model	
Nastos et al.^b^ (2008) [49]	Athens,	Children aged	Poisson GLM	Monthly mean	Monthly number of	β = 0.0273, *P*<0.0001	No adjustments	76.92
	Greece	0–4 years		relative	childhood asthma	( 95% CI: 0.0264–0.0282)	were conducted	
	1978–2000			humidity	admissions(CAA)			
Lee et al.^b^ (2008) [54]	Taiwan	317,926	Cross-sectional	Monthly mean	Prevalence of	1)Boys: RR = 1.15, *P*<0.05	Adjusted for age,	78.13
	1995–1996	children aged	study and Logistic	lowest relative	flexural eczema	( 95% CI: 1.04–1.27)	parental education	
		mostly	regression	humidity		2)Girls: RR = 1.22, *P*<0.05	level, asthma and	
		between 12–14				(95% CI: 1.08–1.38)	active smoking habit	
du Prel et al.^b^ (2009) [40]	Mainz,	3044 children	Multiple time	Mean	Hospitalization	Human rhinovirus :	Correlations among	77.78
	Germany	aged 0–16 years	series analysis	relative	frequency due to	β = 0.218, *P* = 0.002	meteorological	
	2001–2006			humidity	acute respiratory tract		parameters were	
					infections (ARI)		controlled for	
Murdoch and Jennings (2009) [41]	Christchurch,	Children aged	Ecological study,	Monthly	Incidence rates of	IRR = 1.87, *P* = 0.04	No adjustments	80.77
	New Zealand	<5 years	Negative binomial	occurrence of	invasive pneumococcal	(95% CI: 1.02–3.45)	were conducted	
	1995–2006		regression analysis	daily mean 9am	disease(IPD) for the			
				humidity>75%	1-month lag			
Mireku et al.^b^ (2009) [51]	Detroit,	25,401 children	Time–series study,	Intraday	The counts of	β = 0.10, *P* = 0.04	Adjusted for	73.08
	America	aged 1–18 years	Generalized	humidity	asthma admissions		seasonality, air	
	2004–2005		additive model	change	1-day later		pollution, and	
			(GAM)				aeroallergen exposure	
Charland K. M. et al.^b^ (2009) [57]	37 paediatric	63,334 children	Bayesian	Daily mean	The peak week	β = −0.085,	Population type,	74.19
	hospitals in	aged 0–18 years	hierarchical	dew point	of influenza visits	(95% CI: −0.20 to 0.031)	latitude, longitude,	
	USA		models				solar radiation and	
	2000–2005						temperature	
							were considered	
García-Marcos et al. (2009) [50]	Coastal areas,	Children aged	Poisson regression	Annually	Adjusted prevalence	1) 6–7 years old: PR = 1.28	No adjustments	81.48
	Spain	6–7 years and	model	mean	ratios (PR) of current	( 95% CI: 1.12–1.46)	were conducted	
	1994–2002	13–14 years		relative	wheeze per 10%	2) 13–14 years old: PR = 1.25		
				humidity	increase in humidity	( 95% CI: 1.13–1.39)		
								
Connelly et al.^b^ (2010) [59]	Kansas,	25 children	Multilevel model	Daily thrice	Probability of	β = 0.02, *P* = 0.01;	Controlling for	74.07
	America	aged 8–17 years	analyses	relative	headache occurrence	OR = 1.02	changes in the child's	
	2007–2008			humidity			negative affect	
Chou et al.^b^ (2010) [30]	Taiwan	290331 children	Time-series	Monthly mean	Monthly morbidity	β = −0.033, *P* = 0.004	Adjusted for	84.62
	1996–2007	aged 0–14 years	study, Poisson	relative	of diarrhea (no lag)		seasonality, long-term	
			regression	humidity			trends, lag effects	
							and autocorrelation	
Tchidjou et al.^b^ (2010) [42]	Yaoundé ,	1306 children	Negative	Daily	Frequency of	RR = 1.22	Adjusted for	80.00
	Cameroon	aged 0–18 years	binomial	relative	hospitalization for acute	(95% CI: 1.07–1.40)	seasonality and	
	2006–2007		regressions	humidity	respiratory infections		annual trend	
Nascimento-Carvalho et al.^b^ (2010) [23]	Salvador,	184 children	Correlation	Monthly mean	Infections caused by	1) Overall viral infections:	No adjustments	72.00
	Brazil	aged 0–5 years	analysis	relative	different aetiological	r = 0.6, *P* = 0.006	were conducted	
	2003–2005			humidity	agents	2) Chlamydia trachomatis:		
						r = −0.5, *P* = 0.02		
Konstantinou et al.^b^ (2011) [46]	Norwich, UK;	56624 children	Poisson	Monthly mean	Monthly incidence	1) Norwich: IRR = 0.972	Adjusted for	69.23
	Heraklion,	aged 0–14 years	regression model	relative	rate of acute	(95% CI: 0.959–0.985)	seasonal effect	
	Greece			humidity	urticarial	2) Heraklion: IRR = 1.035		
	2005–2007					(95% CI: 1.015–1.056)		
								
Zacarias, Majlender^b^ (2011) [29]	Maputo,	60943 infants	Bayesian	Monthly mean	Malaria incidence	RR = 0.049	The spatial and	73.08
	Mozambique	aged 0–4 years	hierarchical	relative		(95%CI: 0.03048-	temporal correlations	
	2007–2008		models	humidity		0.06531)	were considered	
Loh et al.^b^ (2011) [43]	Singapore	42155 children	Time–series	Weekly	Admissions due to	1) URTI: β = −0.228,	Select model with	84.00
	2003–2008	almost aged 0–5	study, and	relative	upper and lower	*P* = 0.0200;	the lowest Akaike's	
		years	ARIMA model	humidity	respiratory tract	2) LRTI: β = −0.760,	Information Criterion	
					infection ( URTI	*P*<0.0001		
					and LRTI)			
Onozuka, Hashizume^b^ (2011) [25]	Fukuoka,	67000 children	Time–series study,	Weekly mean	Weekly number of	Percentage increase:	Adjusting for	88.46
	Japan	aged<15 years	Negative binomial	relative	mumps cases per 1%	1.4% (95% CI: 0.5–2.4)	temporal, seasonal and	
	2000–2008		regression	humidity	increase in humidity		inter-annual variations,	
					for 0–2 weeks lag		and temperature	
Arnedo Pena et al.^b^ (2011) [56]	Spain	24301 children	Logistic regression,	Annually	Prevalence rate	β = −0.00334,	Prevalence rate of	85.19
	1971–2005	aged 13–14	Multiple regression	mean relative	of asthma	*P*<0.0001	asthma and collinearity	
		years	models	humidity			were adjusted	
Onozuka, Hashizume^b^ (2011) [26]	Fukuoka,	61736 children	Time-series study,	Weekly mean	Percentage increase in	Percent change: 4.7%	Adjusting for seasonal,	85.71
	Japan	aged 0–4 years	Negative binomial	relative	number of hand, foot,	(95% CI: 2.3–7.1)	long-term trends, and	
	2000–2010		regression	humidity	and mouth disease per		inter-annual variations	
					1% increase in humidity			
					with 0–3 weeks lag			
Onozuka, Hashizume^b^ (2011) [27]	Fukuoka,	423142 children	Time-series study,	Weekly mean	Percentage increase in	Percent change: 3.9%	Adjusting for seasonal,	88.89
	Japan	aged<15 years	Negative binomial	relative	number of infectious	(95% CI: 2.8–5.0)	inter-annual, and	
	2000–2008		regression	humidity	gastroenteritis per 1%		temperature variations	
					decrease in humidity			
					for lags of 0–6 weeks			
Wang et al.^b^ (2012) [58]	15 states	1459 children	Multivariable	Daily mean	Hospital admission	OR = 0.9 , *P* = 0.04	Adjusting for pressure	76.67
	in USA	<2 years of age	logistic regression	dew point		(95% CI: 0.8–0.996 )	altitude, wind speed,	
	2004–2006						and temperature	
Khor et al.^b^ (2012) [39]	Kuala	10269 children	Multiple	Monthly mean	Monthly number of	β = −1.070,	No adjustments	74.07
	Lumpur,	aged 0–5 years	regression	relative	respiratory syncytial	*P*<0.001	were conducted	
	Malaysia		analysis	humidity	virus (RSV) cases			
	1982–2008							
Chang et al.^b^ (2012) [24]	Taiwan	1914 children	Case-crossover	Daily mean	Incidence of	IRR = 1.08, *P*<0.001	Adjusted for seasonal,	68.97
	1998–2008	aged<15 years	study, and	relative	Enterovirus 71	(95% CI: 1.05–1.10)	temporal trends and	
			Logistic regression	humidity	infections		annual variations	
Turkish Neonatal^b^ (2012) [37]	Turkey	3464 children	Correlation analysis	Monthly mean	Hospitalization with	r = 0.627, *P*<0.001	Trends and seasonality	73.08
	2008–2010	aged<2 years	and Spearman	relative	respiratory syncytial		were described	
			correlation test	humidity	virus infection			

**Abbreviations**: GAM, generalized additive model; GLM, generalized linear models; ARIMA models, autoregressive integrated moving average modeling; r, correlation coefficient; β, regression coefficient; R^2^/r^2^, coefficient of determination; RR, relative risk; OR, odds ratio; IRR, incident rate ratio; CI, 95% confidence interval; RSV, human respiratory syncytial virus; IA, influenza virus A.

a These included articles are ordered by the date of publication and the first word of their titles.

b In these studies there is more than one meteorological factor that was investigated and analyzed, respectively or simultaneously.

[N]: N is the specific citation number of the related study included in the present review.

### Gastrointestinal and other infectious diseases

Studies found that ambient humidity plays an important role in the occurrence and prevalence of pediatric infectious diseases. A Brazilian study found that among the children hospitalized with community-acquired pneumonia, overall viral and Chlamydia trachomatis infections correlated with monthly mean relative humidity [23]. In Taiwan, a study identified a positive association between daily mean relative humidity and the incidence of enterovirus 71 (EV71) infections among children under 15 years of age (IRR = 1.08, 95% CI: 1.05–1.10), with seasonal and annual trends being controlled [24].

In Japan, one study indicated that for each 1% increase in the weekly mean relative humidity, the number of mumps cases increased by 1.4% (95% CI: 0.5–2.4%) among children [25]. In another Japanese study, the weekly number of hand, foot, and mouth disease cases increased by 4.7% (95% CI: 2.3–7.1%), in children between 0–4 years of age, for each 1% increase in relative humidity [26]. An additional study investigated the relationship between weather variability and infectious gastroenteritis (IG) in children. However, the results indicated that for every 1% decrease in weekly mean relative humidity, the number of IG cases increased by 3.9% (95% CI: 2.8–5.0%) [27].

Existing studies suggest that ambient humidity has a significant impact on malaria risk and diarrhoeal diseases among children. A prior study found that the clinical malaria risk increased non-linearly with the increase in mean relative humidity of the previous month [28]. A Mozambican study reported that humidity was a significant predictor of malaria incidence, with every additional 1% increase in relative humidity level leading to a 5% increase in risk of malaria incidence [29]. In Taiwan, a study examining the relationship between climate variations and diarrhea-associated morbidity found that there was a significant negative correlation between relative humidity and the morbidity of diarrhea in 0- to 14-year-old children (β = −0.033, *P* = 0.004) [30]. Another study performed in Peru indicated that an increase of 1% in mean relative humidity was associated with a 3% decrease in the number of diarrhoeal admissions [31]. The results of an Australian study showed that higher humidity in the previous week was associated with a decrease in rotavirus diarrhoeal admissions among children hospitalized for rotavirus diarrhoea after controlling for season [32].

### Respiratory system diseases

Many pediatric respiratory system diseases are sensitive to relative humidity. An Argentinean study reported that respiratory syncytial virus (RSV) and influenza virus A had a positive correlation with mean monthly relative humidity among children hospitalized due to acute lower respiratory viral infections [33]. Another study found that low absolute humidity presented a protective effect on the admission of infants who had lower respiratory tract infection (LRTI) due to RSV [34]. In a study conducted in Jordan, a significant positive correlation between the prevalence of RSV infection and relative humidity was identified among hospitalized children [35]. A similar study in Indonesia indicated that for children hospitalized with clinical pneumonia, a 1% rise in mean relative humidity was associated with a 6% increase in RSV cases [36]. An additional study of children hospitalized with LRTI in Turkey reported that the number of RSV infection cases positively correlated with relative humidity, similar to results reported by Noyola and Mandeville for Mexico [37], [38]. Another retrospective study was performed in Malaysia to investigate the viral etiology of children who were admitted with viral respiratory tract infections. However, the results showed that monthly RSV cases were inversely correlated with relative humidity [39].

In Germany, a study found that human rhinovirus positively correlated with relative humidity among children who were hospitalized with acute respiratory tract infections (ARIs) [40]. Another study in New Zealand showed that the number of days per month with a mean 9 AM humidity >75% positively correlated with the incidence of invasive pneumococcal disease in children [41]. The results of a study in Cameroon showed that high relative humidity was directly associated with an increase in the frequency of hospitalization from ARIs [42]. However, in one Singapore study, of 42155 children presenting in the hospital with one of five common paediatric diseases, both upper respiratory tract infections and LRTI were negatively correlated with relative humidity [43].

### Dermatopathy and pediatric allergic diseases

Childhood asthma and other pediatric allergic diseases are important adverse consequences of humidity. In Greece, the results of a study showed that relative humidity was an implicated meteorological variable for younger asthmatic children [44]. A study performed in fourteen cities in Turkey found that asthma, wheezing and allergic rhinitis were associated with higher mean yearly outdoor humidity among school children [45]. Another study reported an interesting finding that relative humidity was significantly associated with the incidence of acute urticaria in childhood. However, the humidity accounted for different effects in the two study cities. Specifically, decreasing incidence was found for humidity increases in Norwich, Britain, while the inverse was observed in Heraklion, Greece [46].

In one Turkish study of 21 bronchial asthma children, results indicated that increasing relative humidity leads to an increase in total daily complaints of asthma patients [47]. A study reported that winter humidity was positively associated with the prevalence of asthma in Taiwanese middle-school students [48]. A positive relationship between mean monthly relative humidity and childhood asthma admissions (CAAs) was reported in Greece. For this study, a 10% increase in humidity was related to a 31% increase in the probability of having a CAA [49]. In addition, an analysis, carried out exclusively within the coastal region of Spain, showed that relative humidity constituted a significant risk factor for wheezing in both the 6–7 and 13–14 age groups [50]. A retrospective study performed in America indicated that a 10% intraday increase in humidity one or two days before admission was associated with approximately 1 additional emergency department visit for asthma [51].

On the contrary, a study in Taiwan indicated that relative humidity was negatively correlated with asthma admission rates of pre-school and school-age children [52]. In Western Europe, a positive relationship between the lowest monthly mean relative humidity and prevalence rates of asthma symptoms was found among children [53]. Another similar study conducted among school children in Taiwan indicated that the lowest monthly mean relative humidity is positively related to eczema [54]. An additional study conducted in Japan reported that the number of emergency visits for childhood asthma per night increased significantly when climate conditions showed a rapid decrease from higher humidity [55]. A study in Spain also showed that relative humidity was negatively associated with asthma in the 13–14 age group [56].

### Others

Dew point, another measure of ambient humidity, is the temperature at which water vapor in the air becomes saturated and water droplets begin to form. To date, relative humidity and dew point are two widely used indicators for the amount of moisture in air. Accordingly, some other studies also investigated the association between ambient humidity and child health by choosing dew point as the indicator. A negative association between the peak week of child-related influenza visits and dew point was reported by Charland and colleagues, although the relationship was not significant [57]. In another study, Wang et al. found that a higher dew point was associated with a lower risk of hospital admission among children [58].

In addition, ambient humidity may also play a role in other diseases among children. For instance, the results of an American study showed a positive association between relative humidity and the likelihood of headache among children [59].

## Discussion

The purpose of the present systematic review was to summarize key findings related to the effects of ambient humidity on child health, and identify the relationship between humidity exposure and common pediatric diseases. The systematic review of the 37 included publications performed herein demonstrates that ambient humidity generally plays a significant role in the incidence and prevalence of childhood climate-sensitive diseases, especially for gastrointestinal, respiratory system, and allergic diseases. Considering the expected continuation of climate change, the adverse influence of ambient humidity on children may become increasingly serious. However, some inconsistencies or even contradicting directions and magnitudes of the effects were also observed.

Almost 73% of the final included studies were conducted in high-income countries or regions, indicating that more attention should be paid to the developing countries of Asia and Africa. Because of low-income, a large population and poor infrastructure, people in these countries are usually more vulnerable to climate-sensitive diseases. The results of the quality assessment indicate that the combined application of advanced statistical methods, such as time-series analysis combined with Poisson regression, usually tends to provide more reliable results (higher quality ratings), since adjustments could be made for potential confounders, including long-term trends, seasonality and air pollution.

### Impact of ambient humidity on child health

Because of their physiologic, metabolic and cognitive immaturity, and greater sensitivity to certain exposures, children are often the most vulnerable to adverse health effects from environmental hazards [14], [16]. In addition, more expected future years of life will expose them to newly developing or worsening environmental hazards [7]. In general, child vulnerability to environmental hazards (e.g. humidity) can be potentially explained by their unique modality of physiological, metabolic, behavioral, and self-care ability [60], [61]. Therefore, with global climate change, children are likely to suffer disproportionately from weather factors [60], [62]. It is thought that if children are adversely affected by meteorological factors (e.g. humidity), their long-term health may also be adversely impacted [63].

Findings from the present review suggest that the occurrence and prevalence of childhood climate-sensitive infectious diseases are significantly associated with ambient humidity. It is predicted that climate change will lead to more frequent and abundant rainfall, increasing temperature and higher concentrations of water vapor, which will cause favorable conditions of ambient humidity and concomitantly favor increases in the occurrence and development of infectious diseases [3], [4], [10]. For example, ambient humidity together with temperature can influence the population dynamics of vectors, persistence and survival of pathogens, seasonal activity of microorganisms, and duration of the life cycle of parasites, and therefore impact the occurrence and prevalence of various infectious diseases [10], [63], [64].

Ambient humidity can affect tick abundance and seasonal tick activity. It has been reported that the host seeking activity of *I. ricinus* depends on the relative humidity, and below a relative humidity threshold of 86%, the tick dehydrates and cannot continuously seek a host [65], [66]. In Sweden, a study found that the number of days with levels of relative humidity above 86% correlates positively with the incidence rate of erythema migrans [67].

For gastrointestinal infectious agents such as rotaviruses and EV71, changes in ambient humidity have been reported to facilitate viral resistance, thereby enhancing the virulence and increasing the efficiency of transmission through contaminated air, fomites and environmental surfaces [68], [69]. A laboratory-based study suggested that the stability of enteroviruses in external environmental conditions is dependent on temperature and humidity, and another study reported that raising the relative humidity to 80% results in a rapid loss of infectivity, suggesting that humidity might be an important environmental determinant of rotavirus infectivity [68], [70]. This perhaps can explain why the relative humidity has a linear inverse relationship with the number of cases of rotavirus diarrhea previously reported in a study of 3115 rotavirus diarrhoea cases in 0- to 2-year-old Bangladesh children [71]. However, a study performed in China suggested that the number of hand, foot, and mouth disease infections is positively correlated with ambient humidity [72].

Malaria is a climate-sensitive vector-borne illness. Meteorological factors (e.g. temperature, humidity) have considerable impact on *Anopheles* vector abundance and the extrinsic cycles that the parasites perform inside mosquitoes. Thus, meteorological factors may affect malaria incidence and constitute driving forces for malaria epidemics [73]. A study in China suggested that moderate temperature and relative humidity increase the longevity of the adult mosquito so that it can transmit infection for a longer period of time [74]. In India, a study found a higher positive correlation between monthly malaria parasite incidence and ambient humidity [75].

Many studies demonstrate the effects of relative humidity on pediatric respiratory tract infections. In tropical and subtropical areas (e.g. Singapore, Malaysia), it has been suggested that high humidity and temperature prolong the activity and stability of microorganisms in large-particle aerosols, and permit year-round transmission of the microorganisms [5], [76]. A study reported that relative humidity is positively associated with RSV activity, with more RSV infection occurring when humidity increases [77]. However, a converse conclusion was found in an Argentinean study, which concluded that humidity provides a protective effect against respiratory infections [78].

The possible effects of ambient humidity on childhood asthma and other allergic diseases are of particular concern due to this population's greater susceptibility. Meteorological factors (humidity, temperature) can influence both biological and chemical components of air and might induce negative effects on respiratory allergic diseases. With climate change, ambient humidity is likely to impact asthma, hay fever and other allergic diseases via its influence on the concentration and distribution of pollens, mould spores, house dust mites, and other aeroallergens [79], [80]. Moreover, changes in the outdoor humidity, resulting from climate change, is important not only because of its own effects, but also because of its influence on indoor humidity, which results in infestation of homes with house dust mites, growth of fungal spores and home dampness, and is associated with the incidence of allergic diseases [12], [79], [81]. A study conducted among school children in Spain indicates that humidity significantly influences the occurrence of atopic eczema [82]. Youssefagha and colleagues indicate that upper-air relative humidity ≥50% and higher dew point (>−1.9) are significantly associated with exacerbation of asthma among children [83]. Another study performed in Japan also suggests that asthmatic children frequently visit the emergency department during early mornings with high absolute humidity [84].

However, in this review, some studies also report a negative association between ambient humidity and pediatric allergic diseases [52], [56]. The reasons for the inconsistencies among different studies are possibly due to regional differences and local characteristics, such as geographical environment, type of climate, vegetation condition, air quality, socio-economic status, and the acclimatization and adaptation of the local populations [63], [79], [80]. Additionally, study design, subgroups of children and method of analysis may also contribute to the controversies.

As mentioned above, different studies may present inconsistencies in the direction and magnitude of the effects of ambient humidity, with other studies even concluding that the occurrence of relevant climate-sensitive diseases appears to be unrelated to humidity [36], [39], [85], [86]. This discrepancy can be explained by the following possible reasons: firstly, relative humidity can affect microorganisms in different ways. Some microorganisms tend to be more invasive in increased humidity, while others develop better in lower humidity [10]. Similar effects of ambient humidity on the concentration and distribution of aeroallergens have also been reported [87]. Secondly, there is a geographical, meteorological and temporal heterogeneity in the different studies. The occurrence of different effects suggests that geographical sites, regional variability and acclimatization of the local population may play a role in determining the exposure–response relationship. Thirdly, among various studies, whether or not one takes into account potential confounding factors and what kind of confounder is adjusted for existing differences, can make a difference in the conclusions. Moreover, even mutual confounding factors among different studies are likely to vary between regions, which implies that regional differences and local characteristics may modify the effects of weather factors [25], [27], [71]. Lastly, the date of the study conducted, the limited study duration and participants, and the different age groups of the study population may also play a role in the discrepancy. Future research taking these factors into account will be appreciated.

### Strengths and limitations

This systematic review begins to address the current knowledge gap in the relationship between ambient humidity and child health. This is the first attempt to synthesize, using a systematic approach, the existing evidence of humidity-related health outcomes, across different countries, among children between 0–18 years of age. According to **Table 2**, almost all of the final included journal articles (34/37) were published after the year 2000, and were of relatively high reporting quality (the majority of records having a reporting quality above 70%). The findings in **Table 2** suggest that ambient humidity has been playing a significant role in weather-related health outcomes. Unfortunately, children are usually the most susceptible subpopulation. The present review is crucial because this literature provides details of the current state of studies that examine the effects of humidity on child health and proposes directions for the future research.

Despite targeting the reporting quality of observational studies, many items of the STROBE checklist were no doubt selected due to evidence of association with susceptibility to bias. Thus experts have suggested that the STROBE statement provides a suitable starting point for development of a quality assessment tool [20], [88]. Just as the MOOSE (Meta-analysis Of Observational Studies in Epidemiology) guidelines are being used to assess quality, although they were actually developed as reporting guidelines, in this review, we tried to describe the reporting quality of the final identified articles by using an adapted version of the STROBE checklist [18], [89]. After the quality assessments, studies that applied credible and convincing statistical methods and outcome measures often obtained higher quality scores at the same time (**Table 2**), indicating that the adapted STROBE checklist can describe the reporting quality of the final identified articles well. This novel quality assessment tool is innovative and potentially ground-breaking in terms of its contribution to relevant environmental and meteorological literature reviews [22].

However, regarding the results of this systematic review, some limitations should be acknowledged. First, this review is limited by its emphasis on papers published in English in peer-reviewed journal databases, so we may have missed potentially useful studies. Second, despite the highly sensitive and comprehensive nature of the search strategy employed, it is unlikely that all existing studies have been identified, and publication bias cannot be entirely excluded. Third, the adapted version of the STROBE checklist was initially implemented to describe the reporting quality of weather-related studies, so the validity and reliability of the checklist for our purposes have not been completely tested and formally evaluated. However, in this review, the tool was used only to describe the reporting quality, and the quality scores were not included as part of the relevance criteria. Fourth, it has been reported that temperature and relative humidity are not independent of one another, so the effects of relative humidity may be confounded by temperature. However, in our review, the majority of studies adjusted for potential confounding factors (e.g. temperature) when analyzing the impact of humidity on child health, and studies that conducted no adjustments were given lower quality ratings. Additionally, relative humidity is a popular indicator that has been widely used in current research and meteorological monitoring. Therefore, it is helpful and of practical significance to choose relative humidity rather than absolute humidity or water vapor pressure as the exposure variable. Finally, the data extraction and quality assessment were not performed independently by more than one reviewer, which may have led to subjective errors and introduced bias. However, in the present review, due to the explicit inclusion criteria, specially pre-designed data extraction form, definite exposure variable and health outcome, and the adapted quality assessment tool, the authors were able to ensure the integrity of the review, and to be unbiased during the conduction of assessment.

## Conclusion

In summary, this study demonstrates that due to various inherent and unique characteristics, children are often susceptible to ambient humidity. We find that with climate change, there is a significant impact of humidity on child health, especially for climate-sensitive infectious diseases, diarrhoeal diseases, respiratory system diseases, and pediatric allergic diseases. However, the humidity-related effects on child health are various and complex. Ambient humidity continues to impact children unequally and in different ways, with impacts ranging from direct and indirect, and negative and positive. In this review we find that the influence of humidity on child health involves heterogeneous and even converse effects among different study regions, research designs, health outcomes, adjustments of potential confounding factors (e.g. air pollution, seasonality, interaction with other meteorological factors), and subgroups of children, which suggests that further studies focusing on the association between ambient humidity and child health are needed to deal with the aforementioned problems.

Current increasing interest in the relationship between climate change and child health may provide an ideal opportunity to employ various epidemiological and statistical methods to further explore the impact of ambient humidity on child health. Such research and epidemiologic evidence can inform the local health officials and policy-makers to make and implement necessary preparations (mitigation and adaptation), ultimately contributing to better health outcomes for a humidity-impacted population.

## Supporting Information

S1 Checklist PRISMA checklist.
(DOCX)
